# Genotoxic risk assessment and mechanism of DNA damage induced by phthalates and their metabolites in human peripheral blood mononuclear cells

**DOI:** 10.1038/s41598-020-79932-5

**Published:** 2021-01-18

**Authors:** Paulina Sicińska, Katarzyna Mokra, Katarzyna Wozniak, Jaromir Michałowicz, Bożena Bukowska

**Affiliations:** 1 Department of Biophysics of Environmental Pollution, Faculty of Biology and Environmental Protection, University of Lodz, Pomorska Str. 141/143, 90-236 Lodz, Poland; 2 Department of Molecular Genetics, Faculty of Biology and Environmental Protection, University of Lodz, Pomorska Str. 141/143, 90-236 Lodz, Poland

**Keywords:** Cell biology, Genetics, Molecular biology

## Abstract

The human genome is persistently exposed to damage caused by xenobiotics, therefore the assessment of genotoxicity of substances having a direct contact with humans is of importance. Phthalates are commonly used in industrial applications. Widespread exposure to phthalates has been evidenced by their presence in human body fluids. We have assessed the genotoxic potential of selected phthalates and mechanism of their action in human peripheral blood mononuclear cells (PBMCs). Studied cells were incubated with di-n-butyl phthalate (DBP), butylbenzyl phthalate (BBP) and their metabolites: mono-n-butylphthalate (MBP), mono-benzylphthalate (MBzP) in the concentrations range of 0.1–10 µg/mL for 24 h. Analyzed compounds induced DNA single and double strand-breaks (DBP and BBP ≥ 0.5 µg/mL, MBP and MBzP ≥ 1 µg/mL) and more strongly oxidized purines than pyrimidines. None of the compounds examined was capable of creating adducts with DNA. All studied phthalates caused an increase of total ROS level, while hydroxyl radical was generated mostly by DBP and BBP. PBMCs exposed to DBP and BBP could not completely repair DNA strand-breaks during 120 min of postincubation, in opposite to damage caused by their metabolites, MBP and MBzP. We have concluded that parent phthalates: DBP and BBP caused more pronounced DNA damage compared to their metabolites.

## Introduction

Human genome is often a target for various xenobiotics. Its instability may cause many diseases^1–4^. DNA damage may be caused by environmental factors, intracellular metabolic processes, DNA recombination and replication errors. The most common DNA lesions are single- and double-strand breakages, loss of a nitrogen base, oxidative damage and DNA adducts formation^5,6^. Numerous xenobiotics, including endocrine disrupting chemicals (EDCs), are able to generate reactive oxygen species (ROS) responsible for oxidative stress. Excess of ROS, and particularly of hydroxyl radical, may lead to DNA damage, and thus may contribute to carcinogenesis^7^.

Phthalates are salts and esters of phthalic acid. They are ubiquitous environmental chemicals well-known as EDCs and peroxisome proliferators (PPs)^8^. Epidemiological studies have indicated a significant exposure of humans to phthalates, which is associated with their softening properties, and therefore common use in food, pharmaceutical and cosmetic industry^9,10^. In Europe, approximately 1 million tons of phthalic acid esters (PAEs) are used annually, and the world annual consumption of those compounds is approximately 30 million tons^11,12^. According to the EU Commission Directive 2018/2005, four phthalates, including DEHP (di(2-ethylhexyl) phthalate), DBP, BBP and DIBP (diisobutyl phthalate), are considered to be harmful for human reproduction (category 1B). Those phthalates must not be contained in products intended for children in the amount of over 0.1% (single nor sum of all contained phthalates) of the overall weight of the product^13^.

Phthalates are not covalently bound to the plastic, and thus they can leak out of various products used every day^14,15^. Phthalates enter humans mostly with food, water, pharmaceutical products and cosmetics. Food is the main source of the exposure of human population to phthalates. It is estimated that food-related exposure to DBP and BBP is 7–10 μg/kg/day^16^. DBP and BBP were detected in the concentrations from 3.287 to 11.083 μg/kg in cooking oil^11,17^, while in dairy products the average content of BBP was approx. 8.4 μg/kg. In pasta, rice, fruits, vegetables, fish and meat DBP and BBP levels reach 300 μg/kg^11,18,19^. Moreover, DBP has been detected in drinking water, bottled water, beer and juices^9,15^ as well as in drug shells, with estimated daily consumption of approx. 233 μg/kg/day^20,21^. Phthalates contained in cosmetics are also able to penetrate humans by skin. DBP and BBP were detected in perfumes, with the maximum concentration of 0.642 μg/mL and 201.724 μg/mL, respectively^22,23^. DBP and BBP have a low potential of bioaccumulation, after entering the organism they are decomposed by lipases and esterases to monoesters, such as mono-n-butylphthalate (MBP) and mono-benzylphthalate (MBzP)^24,25^. Significant levels of DBP and BBP, and their metabolites have been detected in humans (Table 1).

**Table 1:** Concentration range of phthalates and their metabolites determined in human body.

Phthalate	DBP	BBP	MBP	MBzP	References
					
Venous blood	0.051–7.67 μg/mL				^26,27^
Umbrical cord blood	0.0197–5.71 μg/mL	0.0225 µg/mL			^26–28^
Blood serum	0.0008–0.0125 μg/mL 35.1 μg/g	0.0008–0.002 µg/mL			^29,30^
Amniotic fluid			0.0035 μg/mL	0.0002 μg/mL	^28^
Urine	0.0003–0.269 μg/mL		4.6 µg/g creatinine	0.0010–1.02 μg/mL	^26,31–37^
Breast milk	0.022–0.093 µg/mL				^26,37^
Hair samples	2–4 µg/mL	0–6 µg/mL			^38^
Salivia			0.0658 μg/mL	0.3536 μg/mL	^39^

The aim of this study was to assess genotoxic potential and the mechanism of action of selected phthalates in human PBMCs. The phthalates studied (DBP, BBP) are one of the most commonly used plasticizers in the World. European Chemicals Agency (ECHA) identifies DBP and BBP as the substances of very high concern (SVHC)^40^. There is only scarce data on genotoxic potential of DBP and BBP in blood cells, while no information exists regarding genotoxicity of their metabolites. Kleinsasser et al., demonstrated using the comet assay that DBP (354 µmol/mL) caused DNA migration in human lymphocytes^41^.

This information, as well as other reports of genotoxic effects of phthalates on animal cells^42–44^ prompted us to undertake the studies on the mechanism of genotoxic action of selected phthalates: DBP, BBP, as well as to assess genotoxic potential of not previously analyzed phthalates' metabolites (MBP and MBzP) in human PBMCs. We have assessed the sum of DNA single-strand (SSBs) and double-strand breaks (DSBs) in PBMCs. We have also analyzed oxidative damage to purines and pyrimidines. To analyze the mechanism responsible for DNA damage we have assessed the level of total ROS, and the level of highly reactive oxygen species, mostly of the hydroxyl radical. Moreover, we have examined the ability of tested compounds to form adducts with DNA as well as evaluated repair kinetics of DNA strand breaks. The tested compounds were used in the range of the concentrations from 0.1 to 10 µg/mL, which was associated with DBP level determined in human blood (0.0197–7.67 µg/mL)^26,27^. Because the purpose of our study was (among others) to compare toxicity of all examined compounds, the concentrations of other phthalates were adjusted to DBP concentration. The cells were incubated with phthalates for 24 h. The time of incubation was as long as possible to observe changes in PBMCs that did not start to divide yet.

## Results

### DNA damage

Alkaline comet assay enables to determine both SSBs and DSBs formation. Statistically significant increase in DNA damage was induced in PBMCs by DBP and BBP starting from the concentration of 0.5 µg/mL and the tail intensity (TI) was evaluated to be 7.2% and 6%, respectively.

**Figure 1:**
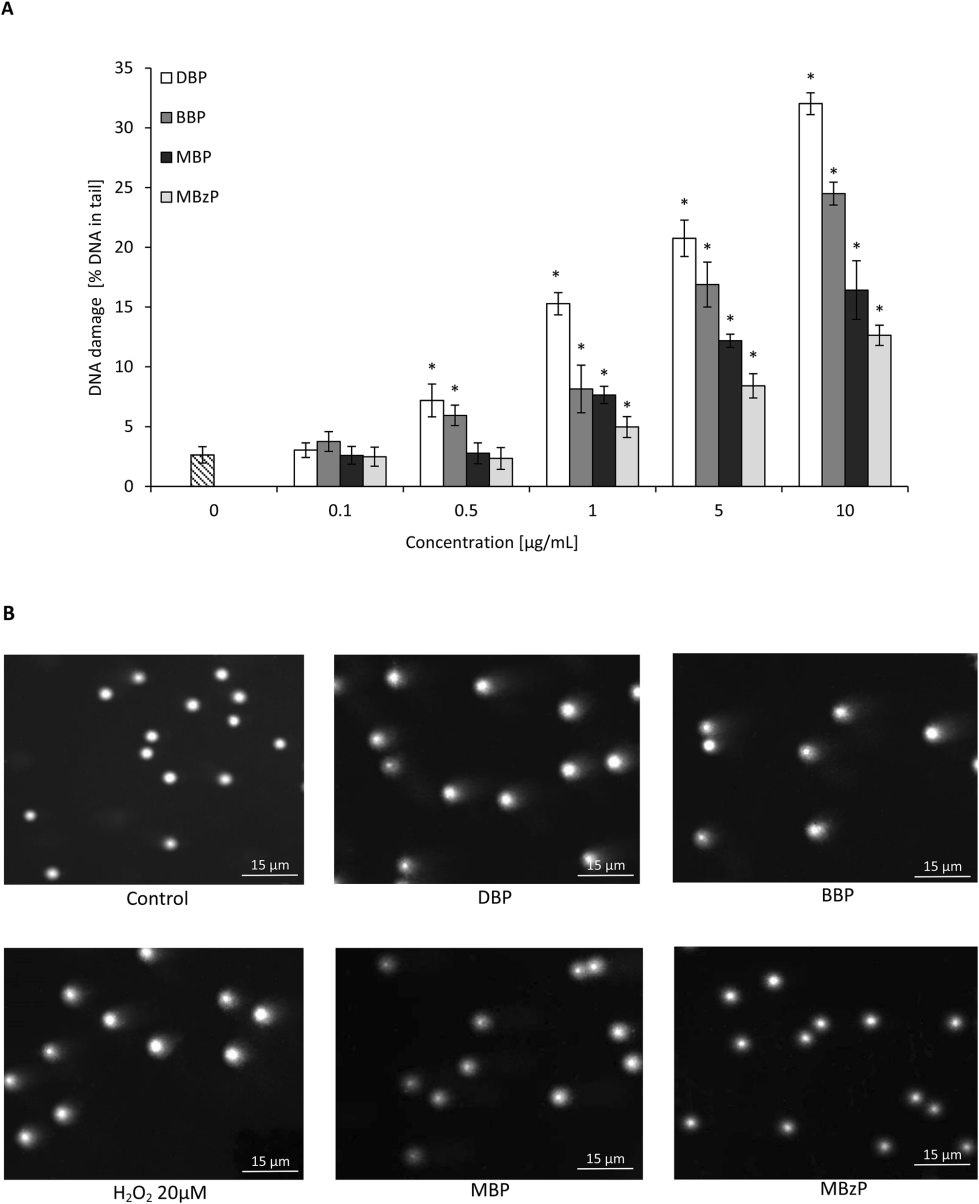
(**A**) The level of DNA strand-breaks in human PBMCs determined by single cell gel electrophoresis (Comet assay). DNA damage in PBMCs was induced by DBP, BBP, MBP and MBzP. The cells were incubated with the compounds examined in the concentrations range from 0.01 to 10 μg/mL for 24 h. DNA damage level was determined as tail intensity (percentage of DNA in comet's tail) by alkaline version of the comet assay. (*) Statistically significant different from control (p<0.05). Each value represents the mean ± SD calculated from 5 individual experiments (5 blood donors). (**B**) Seleted photographs of DNA (comets) of human PBMCs incubated with phthalates in the concentration of 10 μg/mL for 24 h and hydrogen peroxide (20 μM) for 15 min on ice (positive control) (comet assay, alkaline version). The photos were achieved using fluorescent microscope with 200× magnification.

For the highest concentration of DBP and BBP of 10 µg/mL DNA damage was significant—TI was 32% and 24.5%, respectively. For phthalates’ metabolites, the first statistically significant effects were observed for their concentration of 1 µg/mL (TI was 8% and 5% for MBP and MBzP, respectively). However, DNA damage induced by phthalates’ metabolites at 10 µg/mL was approximately half those of parent compounds (Fig. 1).

### Plasmid relaxation assay

**Figure 2:**
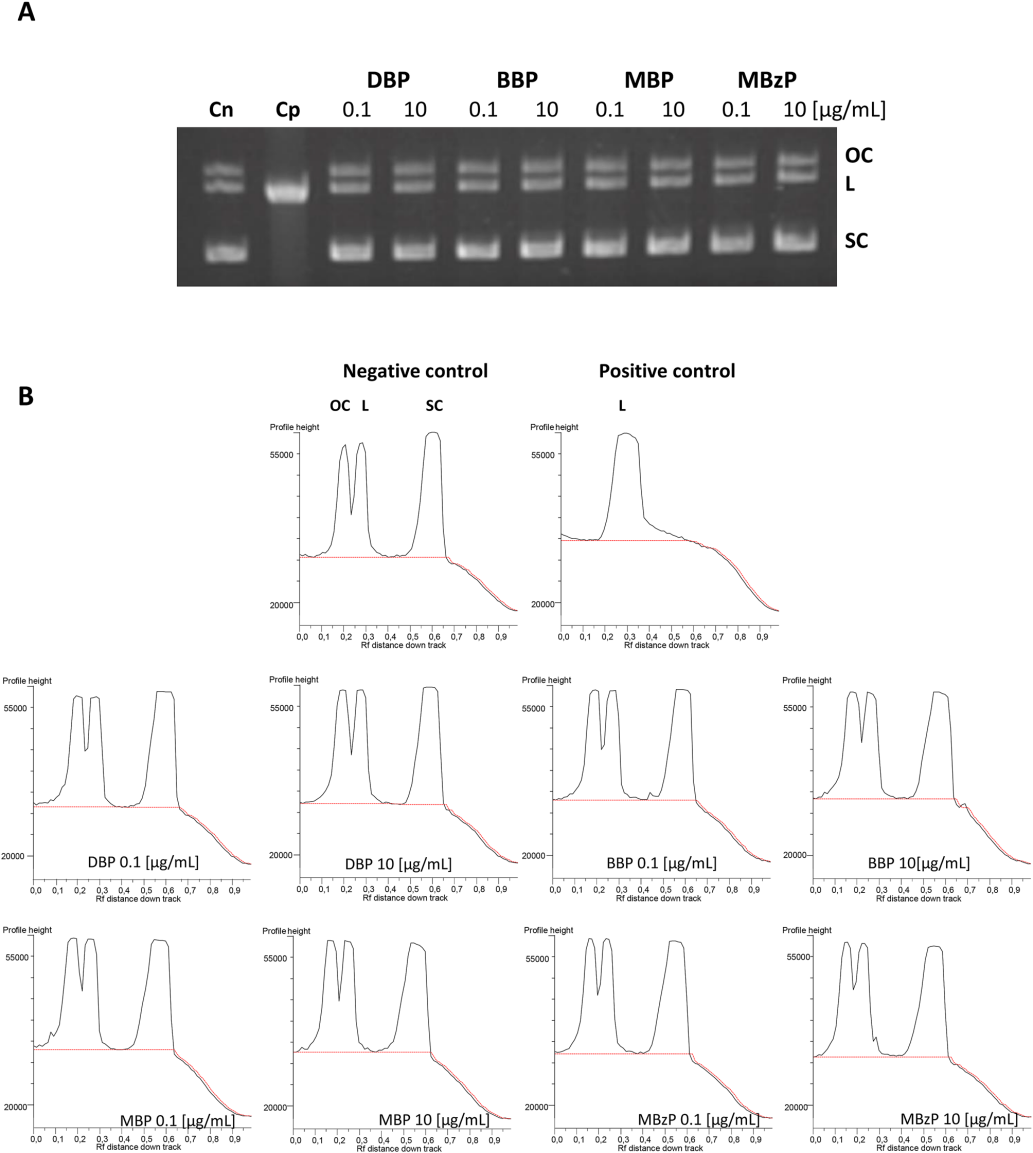
Plasmid relaxation assay. (**A**) pUC19 plasmid DNA was resolved on a 1% agarose gel, stained with ethidium bromide and visualized in UV light. Line 1—negative control (Cn) (pUC19 plasmid); line 2—positive control (Cp) (the plasmid was exposed to 200 µM H_2_O_2_ and 20 µM Fe^+2^ for 20 min on ice, Fenton reaction); lines 3—10 – pUC19 plasmid incubated with DBP, BBP, MBP, MBzP at indicated concentrations. Structural differences between supercoiled (SC), open circular (OC) and linear (L) forms of the plasmid accounted for their different electrophoretic mobility. (B) Densitometric analysis of agarose gel was presented below the gel picture. Open circular (OC) (as a consequence of DNA strand breaks), linear (L) (as a consequence of DNA double strand-breaks) and supercoiled (SC) (undamaged DNA) forms of DNA plasmid are presented as peaks. Densitometric analysis was performed with the GeneTools by Syngene (Cambridge, UK) software.

The results obtained from electrophoretic separation of plasmid DNA showed that neither DBP and BBP nor MBP and MBzP did not bind directly to DNA (Fig. 2A). Also densitometric analysis demonstrated no changes in the amount of various plasmid forms following the phthalate exposure, compared to the positive control. We have therefore assumed that analyzed phthalates were unable to form adducts with DNA (Fig. 2B). Full-length gels are presented in Supplementary Figure S1.

### Oxidative modifications to DNA bases

**Figure 3:**
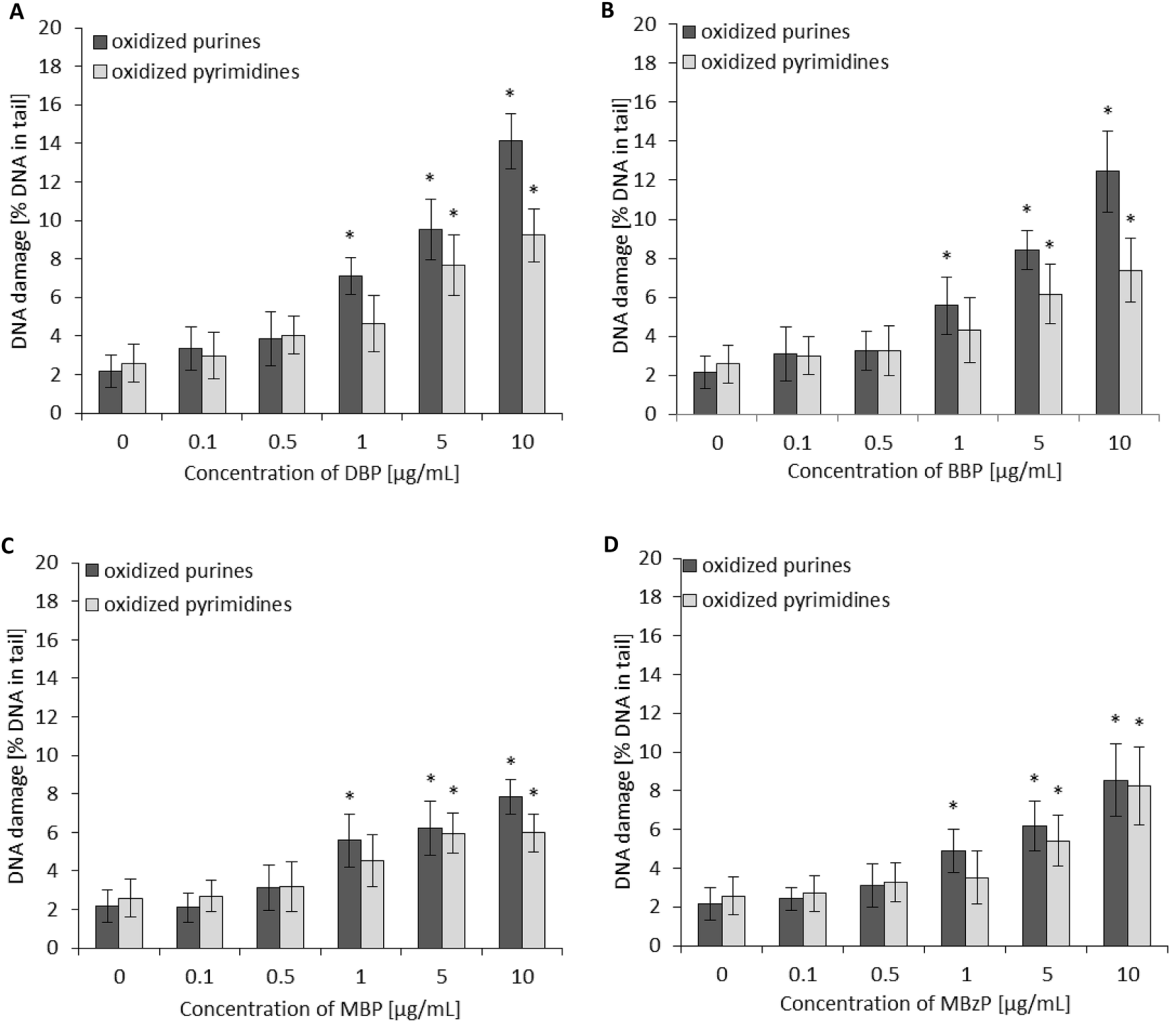
The level of DNA purines and pyrimidines oxidation in human PBMCs (analysis by means of alkaline version of the comet assay with human 8-oxoguanine DNA glycosylase—hOOG1 or endonuclease III–Nth). DNA damage in PBMCs was induced by DBP (**A**), BBP (**B**), MBP (**C**) and MBzP (**D**). The cells were incubated with phthalates in the concentrations range from 0.1 to 10 µg/mL for 24 h. The value of comet tail (damaged DNA) in the presence of either enzyme for different concentrations of phthalates was reduced by the value obtained in comet assay without any enzyme (value for enzymatic buffer for the appropriate concentration of phthalates). (*) Statistically significant different from control (p < 0.05). Each value represents the mean ± SD calculated from 5 individual experiments (5 blood donors).

The level of DNA damage in PBMCs exposed to phthalates and their metabolites followed by the treatment with endonuclease III (Endo III or Nth) or human 8-oxoguanine DNA glycosylase (hOGG1) is shown in Fig. 3. DBP caused an increase of oxidative purine damage with TI level estimated for 7.1%, 9.5% and 14.1% for the concentrations of 1, 5 and 10 µg/mL, respectively. In case of oxidative damage to pyrimidines, statistically significant changes caused by DBP at 5 µg/mL and 10 µg/mL were noted with TI level calculated for 7.7% and 9.2%, respectively.

Slightly lower oxidative damage to purines was noted for BBP. The TI level was calculated for 5.6%, 8.4% and 12.4% for the concentrations of 1, 5 and 10 µg/mL, respectively.

In case of pyrimidines, oxidative changes were evaluated at the level of 6.1 and 7.4%, for the concentrations of 5 µg/mL and 10 µg/mL of BBP, respectively (Fig. 3). Phthalates’ metabolites, MBP and MBzP, caused changes approximately twice as low as induced by parent compounds. They caused an increase of oxidative purine damage with TI levels calculated for 5%, 6% and 8% at the concentrations of 1, 5 and 10 µg/mL, respectively. However, a statistically significant increase of pyrimidine oxidative damage was observed starting from the higher concentration of phthalates metabolites of 5 µg/mL (TI levels of 6% and 5.5% for MBP and MBzP were noted, respectively) (Fig. 3). Summing up, analyzed compounds induced greater oxidative damage to purines compared to pyrimidines.

### Oxidation of H_2_DCFDA

**Figure 4:**
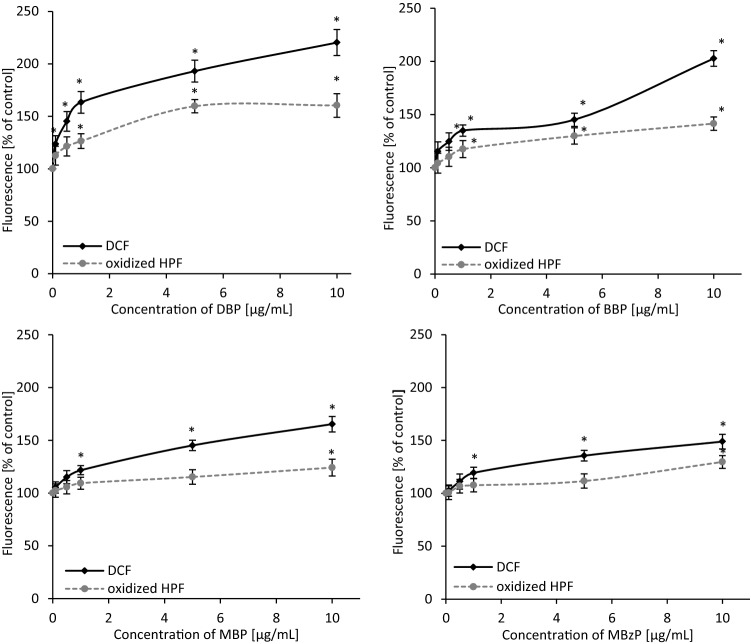
Changes in total ROS (DCF fluorescence) and hydroxyl radical level (oxidized HPF fluorescence) in human PBMCs incubated with DBP, BBP, MBP and MBzP in the concentrations range from 0.01 to 10 µg/mL for 24 h. (*) Statistically significant different from control (p < 0.05). Each value represents the mean ± SD calculated from 5 individual experiments (5 blood donors).

The effect of DBP, BBP, MBP and MBzP on total ROS production in PBMCs was shown as changes in DCF (dichlorofluorescein) fluorescence intensity. The intensity of DCF fluorescence in control PBMCs was referred as 100%. It was demonstrated that analyzed phthalates caused an increase of ROS level. DBP was the first to cause statistically significant changes. ROS level increased starting from DBP concentration of 0.1 µg/mL (by approx. 23%), and then by 45%, 63%, 93% and 120% for the concentrations of 0.5, 1, 5 and 10 µg/mL, respectively. In case of BBP statistically significant changes were observed starting from the concentration of 0.5 µg/mL. Initially they were 24%, followed by 35%, 45%, 102% for respective BBP concentrations of 1, 5, 10 µg/mL. Phthalates' metabolites, MBP and MBzP, caused an increase of total ROS level starting from the concentration of 1 µg/mL (by 22% and 19%, respectively). At the highest concentration of 10 µg/mL the observed changes were 65% for MBP and 49% for MBzP (Fig. 4).

### Oxidation of HPF

Highly reactive oxygen species (mainly hydroxyl radical) level in the cells studied treated with phthalates and their metabolites was determined. Alterations in hydroxyl radical generation in PBMCs were shown as intensity of fluorescence of oxidized HPF (3′-(p-hydroxyphenyl)-fluorescein). The fluorescence intensity of the oxidized probe in control cells was referred as 100%. Parent compounds caused a significant increase of oxidized HPF fluorescence starting from the concentration of 1 µg/mL (by 26% for DBP and by 17% for BBP). Metabolites at higher concentration than their parent compounds increased hydroxyl radical level. MBP and MBzP starting from the concentration of 10 µg/mL caused an increase in oxidized HPF fluorescence intensity by 24 and 29%, respectively (Fig. 4).

### Kinetics of DNA repair

**Figure 5:**
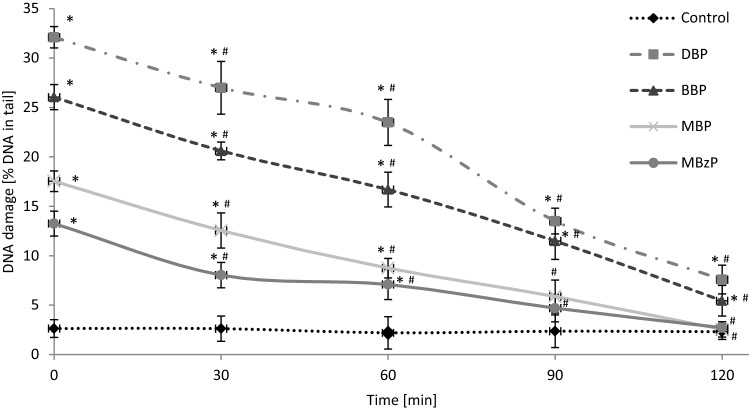
Time course of the repair kinetics of DNA damage (SSBs and DSBs), measured as DNA in comet tail derived from PBMCs treated for 24 h with DBP, BBP, MBP or MBzP in the concentration of 10 µg/mL, then for 2 h in medium deprived of these compounds. (*) Statistically significant different from control (p < 0.05). (^#^) Statistically significant different in comparison to time "0" for individual compound (p < 0.05). Each value represents the mean ± SD calculated from 5 individual experiments (5 blood donors).

Figure 5 shows DNA damage (SSBs and DSBs) in PBMCs exposed to DBP, BBP, MBP or MBzP at 10 µg/mL after 24 h of incubation, as well as DNA lesions after removal of the compounds (time 0 min) and after 30, 60, 90 and 120 min of postincubation of the cells. PBMCs exposed to 20 µM H_2_O_2_ for 15 min on ice (positive control) were able to efficiently repair DNA damage within 120 min (data not shown). We observed efficient repair of DNA damage caused by MBP and MBzP after 90 and 120 min of postincubation—no statistically significant changes were observed after that time in comparison to the control. However, in case of DBP and BBP we observed that PBMCs after 120 min of postincubation could not repair DNA damage completely. DNA damage in that case was 7.6% for DBP, and 5.4% for BBP. Additionally, the efficiency of repair of DNA damage was assessed, which was 83.6%, 88%, 98.6% and 98.8% for DBP, BBP, MBP and MBzP, respectively.

## Discussion

There are numerous contradictory reports of genotoxicity of DBP, BBP and their metabolites, MBP and MBzP, in various types of cells. Those studies fail to explain the mechanism of phthalate-induced DNA damage. The study conducted on earthworms showed that DBP caused DNA damage starting from the concentration of 5 mg/kg after 7 days of exposure^45^. Similar genotoxic effect was observed in rainbow trout cells^42^ and erythrocytes of Nile tilapia after exposure to a sub-lethal concentration of DBP (10 µg/mL for 24 h and 96 h)^44^. In vitro studies carried out on HepG2 cells showed that DBP (10^–8^ – 10^–6^ mol/L) and BBP (2.5 – 250 µM) after 24 h of incubation caused significant increase in DNA damage^43,46^. Moreover, BBP and DBP in the concentration of 1 × 10^–6^ M caused DNA bases lesions in MT3T3-E1 osteoblasts, mouse primary calvarial osteoblasts (COBs), human mucosal cells of the upper aerodigestive tract and human lymphocytes (DBP at 354 µmol/mL)^41,47,48^. On the other hand, there are studies indicating no genotoxic potential of phthalates and their metabolites^23,49–52^. Considering the fact that phthalates are more and more commonly detected in human peripheral blood, umbilical cord blood and blood plasma^26,27,29^, we have assessed genotoxic potential of selected phthalates in human PBMCs and explained the mechanism of genotoxic action of those compounds in this cell type.

On the initial stage we investigated DNA-damaging potential of DBP, BBP and their metabolites: MBP, MBzP in PBMCs using alkaline versions of the comet assay, which allowed to evaluate DNA total strand-breaks (SSBs and DSBs). We observed that parent compounds (DBP and BBP) caused a statistically significant increase of DNA SSBs and DSBs already at the concentration of 0.5 µg/mL, while their metabolites: MBP and MBzP caused statistically significant changes at higher concentration of 1 µg/mL (Fig. 1). Observed changes seem alarming, as they occurred at DBP levels, which have been detected in human peripheral blood (0.051–7.67 μg/mL) and human umbilical cord blood (0.0197–5.71 μg/mL)^26,27,29^. Our study indicated that DBP and BBP had a higher genotoxic potential then their metabolites (MBP, MBzP). The observed findings may be associated with different lipophilicity of those compounds. Parent compounds are characterized by a high logP coefficient, allowing them rapid penetration through membranes, which may explain their genotoxic effect at lower concentrations^53^.

To explain the mechanism of the observed DNA damage, we explored the ability of phthalates and their metabolities to form adducts with DNA. DNA adducts constitute a marker of damage of genetic material, and are formed as a result of the effect of physical factors and electrophilic chemical substances on DNA^54–56^. Using the conformation test, we assessed the effect of tested compounds on the structure of plasmid DNA in order to explain if DNA damage resulted from direct or indirect interaction between DNA and analyzed phthalates. The test demonstrated that none of the analyzed compounds bound directly to DNA (formed adducts) as no formation of linear structure of the plasmid DNA was observed in any case (Fig. 2).

DNA damage of PBMCs exposed to tested phthalates and their metabolites may have also been associated with enhanced production and accumulation of ROS. Some studies have indicated that the exposure of various cell types to phthalates, including DBP is associated with excessive ROS production and potential oxidative damage to DNA^43,57–59^. Excessive accumulation of oxidized DNA bases and their inefficient processing by base excision repair (BER) are among the factors suggested to be contributed to DNA trinucleotide repeat (TNR) expansion, resulting in numerous neurodegenerative diseases^60^. Oxidative DNA damage is also a basis of the ageing process^61^, degenerative diseases, cancer^62^ and atherosclerosis^63^. In this study we assessed the ability of DBP, BBP and their metabolites (MBP and MBzP) to induce oxidative damage of purines and pyrimidines in PBMCs. We observed that parent compounds caused higher oxidation of DNA bases than their metabolites. Moreover, purine oxidation was more pronounced compared to pyrimidine oxidation in PBMCs (Fig. 3.).

ROS are involved in oxidative DNA damage, while hydroxyl radical (HO^·^) seems to be the most important factor^64,65^. The formation of 8-hydroxylated purine lesions in DNA is associated with the mechanism in which the addition of HO^·^ to the C8 of the purine base induces single lesions. Tandem lesions are formed during addition of a peroxyl radical to the C8 of an adjoining purine base, and subsequent decay of the transiently created endoperoxide. Transfer of electron from mostly guanine to peroxyl radicals is responsible for the formation of significant amount of 7,8-dihydro-8-oxo-20-deoxyguanosine, which has been observed in DNA^66^. For that reason, on the next stage of our study we assessed the formation of total ROS and hydroxyl radical in PBMCs exposed to selected phthalates and their metabolites. All analyzed compounds caused production of total ROS, while DBP and BBP demonstrated the highest oxidative potential. However, we observed that a statistically significant increase of hydroxyl radical level was induced only by parent phthalates at the concentrations, which caused oxidative damage to purines (Figs. 4, 3).

**Figure 6:**
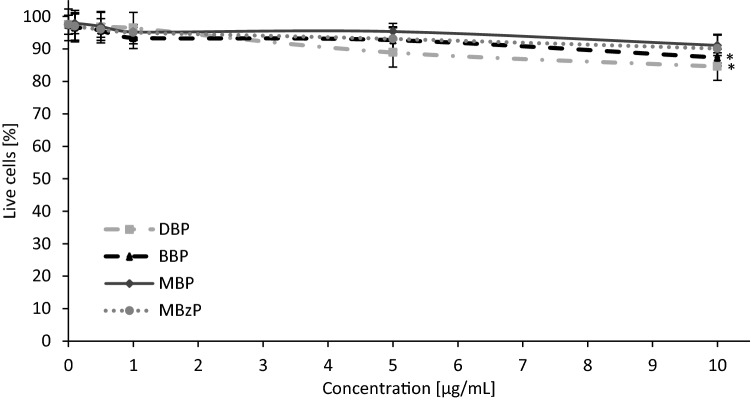
Changes in viability of human PBMCs determined by Trypan Blue dye exclusion test. The cells were incubated with phthalates (0.1–10 µg/mL) for 24 h. (*) Statistically significant different from control (p < 0.05). Each value represents the mean ± SD calculated from 5 individual experiments (5 blood donors).

It is known that DNA is sensitive to various environmental factors affecting its stability, and ultimately responsible for damage to genes. The cellular DNA damage triggers DNA repair, such as the base excision repair to remove oxidatively modified DNA bases, the nucleotide excision repair to rectify gene damage, or the mismatch repair^67,68^. If DNA lesions are not entirely or correctly repaired, there is an increased risk for gene mutations, and cancer may be one of the induced diseases^69^. For that reason, in the next part of our study we assessed the ability of PBMCs to repair (up to 120 min post-incubation) phthalate-induced (10 μg/mL) DNA damage. The analysis demonstrated that damage to DNA caused by phthalates metabolites (MBP and MBzP) was completely repaired. In the case of DBP and BBP at the concentration higher than that found in human blood (max. 7.67 μg/mL) the repair was significant, but not complete. (Fig. 5). DBP and BBP at the highest concentration of 10 μg/mL were cytotoxic to PBMCs (Fig. 6) and probably by this reason there was incomplete repair of DNA damage induced by these compounds. Another reason for the incomplete repair of DNA damage induced by DBP and BBP is that these compounds are much more genotoxic in comparison to MBP and MBzP at 10 µg/mL. It can be assumed, considering the high efficiency of DNA damage repair (83.6% for DBP and 88% for BBP), that if these compounds were used at lower concentrations they would induce DNA damage that would be completely repaired by the cells.

The tested phthalates, and particularly DBP exhibit genotoxic potential in the concentrations that were detected in blood of the general population^26,27^. It may be supposed that similar genotoxic effects (to that observed in our study) may occur in humans chronically exposed to these substances, therefore, it is very important to monitor the concentrations of these toxicants in humans.

The limitation of this study may be age of blood donors. In such studies, it is recommended to obtain blood cells from young donors of 18–35 years old. In our study, we were unable to determine precisely age of blood donors because we purchased the blood from Blood Donation Bank, i.e. personal data of donors (first name, last name, PESEL, sex) were not accessible for us because of protection of personal data. We only received general information (that is contained in our manuscript) that blood donors were non-smoking individuals with no known illness, aged 20–45.

Other limitation may be the range of the concentrations of phthalates used in our study. The compounds were used in the concentrations ranging from 0.1 to 10 µg/mL, which approximately corresponded with DBP concentrations (0.0197–7.67 µg/ml) determined in blood of the general population^26,27^. That is why, the concentrations of other phthalates were adjusted to DBP concentration in order to compare toxicity of all examined compounds.

## Conclusions

It has been shown that the tested phthalates in the concentrations range from 0.1 to 10 µg/mL after 24 h of incubation with PBMCs caused DNA damage (DBP and BBP ≥ 0.5 µg/mL, MBP and MBzP ≥ 1 µg/mL). Analysed compounds caused an increase in ROS (including HO^·^) level and caused oxidative damage to DNA purines (all phthalates tested ≥ 1 µg/mL) and pyrimidines (all phthalates studied ≥ 5 µg/mL). Tested substances did not form adducts with DNA. PBMCs exposed to phthalates and their metabolites (even at relatively high concentration of 10 µg/mL) showed high (but not complete) DNA repair efficiency. First statistically significant changes in the tested parameters were observed at phthalates concentrations relevant for human exposure^26,27^.

The results showed that observed changes were not associated with direct interaction of phthalates studied with DNA, but the most probably they occurred through ROS-mediated effects. Further in vivo studies are needed to confirm our findings in vitro.

## Materials and methods

### Chemicals

Phthalates: di-n-butyl phthalate (DBP), butylbenzyl phthalate (BBP), phthalate metabolites: mono-n-butylphthalate (MBP), mono-benzylphthalate (MBzP) (99–99.5% purity) were bought from Sigma-Aldrich, USA. Fetal bovine serum (FBS), penicylin-streptomycin, low-melting point (LMP) and normal melting point (NMP) agarose and plasmid pUC19 were bought from Sigma-Aldrich (USA). RPMI 1640 medium with L-glutamine and lymphocyte separation medium (LSM) (1.077 g/cm^3^) were purchased in Cytogen (Germany), while other chemicals were bought from Roth (Germany) and POCh (Poland) and were of analytical grade^1^.

### Cells isolation and treatment

PBMCs were isolated from the leucocyte-buffy coat separated from blood bought in the Regional Centre of Blood Donation and Blood Treatment in Lodz, Poland. Blood samples were purchased for research purposes only. Blood was collected from 25 healthy individuals (non-smoking donors with no known illness, aged 20–45). All the procedures related to blood donation were executed at the Regional Centre of Blood Donation and Blood Treatment in Lodz, Poland. The blood donors recruitment was at the Centre, according to national legal procedures and European Union regulations (incl. the regulation (EU) 2016/679 OF THE EUROPEAN PARLIAMENT AND OF THE COUNCIL of 27 April 2016 on the protection of natural persons with regard to the processing of personal data and on the free movement of such data).

PBMCs were isolated using LSM (1.077 g/cm^3^) by centrifugation at 600* g* for 30 min at 20 °C. PBMCs were collected, suspended in erythrocyte lysis buffer (150 mM NH_4_Cl, 10 mM NaHCO_3_, 1 mM EDTA, pH 7.4) and incubated for 5 min at 20 °C. Then, PBS was added immediately, and the cells were centrifuged at 200* g* for 15 min at 20 °C. The supernatant was decanted, and the cells were washed twice with RPMI with L-glutamine and 10% fetal bovine serum (FBS) at 200* g* for 15 min at 20 °C. The cells were resuspended in RPMI medium with L-glutamine, 10% FBS and penicillin–streptomycin (0.5%). The final PBMCs density used in the experiments (after addition of individual phthalate solution) was 1 × 10^6^ cells/mL. The compounds were dissolved in ethanol. The final concentration of ethanol in negative control samples (without phthalate) and samples treated with individual phthalate was 0.2%. The concentration of ethanol used in the experiments (samples) was not toxic for PBMCs as analyzed by all parameters examined^70^.

The final concentrations of the compounds used in the experiments were in the range from 0.1 to 10 µg/mL. The PBMCs were incubated with substances studied for 24 h. The incubation was performed at 37 °C in 5% CO_2_ atmosphere in total darkness^70^. After incubation, the cells were centrifuged, the supernatant was discarded, and the cells were resuspended in RPMI medium. Finally cell viability was determined (Fig. 6). The Trypan Blue dye exclusion test was used to determine the number of viable cells present in a cell suspension^1^.

### DNA damage

Damage to DNA provoked by DBP, BBP and their metabolites (MBP, MBzP) was assessed by means of the single cell gel electrophoresis (comet assay). In this technique, the cells are immersed in low melting point agarose (LMP), placed on microscopic slides, and then lysed. Then, released DNA is submitted to electrophoresis in appropriate conditions. Alkaline comet assay enables to determine both SSBs and DSBs formation. In addition, this method allows to determine alkali-labile sites (ALS), DNA-DNA/DNA–protein cross-links and SSBs associated with incomplete excision repair sites^71,72^.

The comet assay was performed under alkaline conditions according to the procedure of Singh et al.^73,74^ with some modifications^75^. A freshly prepared cells suspension in 0.75% LMP agarose dissolved in PBS was layered onto microscope slides (Superior, Germany), which were pre-coated with 0.5% NMP agarose. Then, the cells were lysed for 1 h at 4 °C in a buffer containing 2.5 M NaCl, 0.1 M EDTA, 10 mM Tris, 1% Triton X-100, pH 10. After cells lysis, the slides were placed in an electrophoresis unit. DNA was allowed to unwind for 20 min in the solution containing 300 mM NaOH and 1 mM EDTA, pH > 13. Each experiment concerning DNA damage included a positive control. Hydrogen peroxide at 20 μM was selected to induce DNA SSBs (the cells were incubated with H_2_O_2_ for 15 min on ice). Electrophoretic separation was performed in the solution containing 30 mM NaOH and 1 mM EDTA, pH > 13 at ambient temperature of 4 °C (the temperature of the running buffer did not exceed 12 °C) for 20 min at an electric field strength of 0.73 V/cm (28 mA). Then, the slides were washed in water, drained, stained with DAPI (2 μg/mL) and covered with cover slips. In order to prevent additional DNA damage, the procedure described above was conducted under limited light or in the dark^1^. The comets were observed at 200 × magnification in an Eclipse fluorescence microscope (Nikon, Japan) attached to a COHU 4910 video camera (Cohu, Inc., San Diego, CA, USA) equipped with a UV-1 A filter block and connected to a personal computer-based image analysis system Lucia-Comet v. 7.3 (Laboratory Imaging, Praha, Czech Republic). Fifty images (comets) were randomly selected from each sample. For one blood donor, three parallel tests with aliquots of the sample of the cells were performed for a total number of 150 comets. A total number of 750 comets (5 blood donors, n = 5) was recorded to calculate mean ± SD^1^. DNA damage level was determined as tail intensity (percentage of DNA in comet’s tail).

### Plasmid relaxation assay

pUC19 plasmid was incubated with DBP, BBP, MBP or MBzP at 0.1 and 10 μg/mL. The plasmid was also exposed to 200 μM of H_2_O_2_ and 20 μM of Fe^+2^ for 20 min on ice (Fenton reaction). We conducted this reaction (positive control) to check the migration of the pUC19 plasmid multimeric forms (supercoiled (SC), open circular (OC) and linear (L)). In this reaction hydroxyl radical was formed that induced DNA strand-breaks and caused the relaxation of supercoiled plasmid. Structural differences between supercoiled, open circular, and linear forms of the plasmid accounted for their different electrophoretic mobility. Plasmid samples at 250 ng were subjected to 1% agarose gel electrophoresis carried out in TAE (Tris–Acetate-EDTA) buffer. The gel was stained with ethidium bromide (0.5 μg/mL), and plasmid DNA was visualized under ultraviolet light (302 nm) and scanned by a CCD camera. Densitometric analysis of the gel was performed with the GeneTools by Syngene (Cambridge, UK) software^76^.

### Oxidative modifications to the DNA bases

Detection of oxidative DNA damage was conducted with the comet assay using endonuclease III (Endo III or Nth) and human 8-oxoguanine DNA glycosylase (hOGG1). Endo III and hOGG1 are capable of converting oxidized pyrimidines and purines, respectively into DNA single-strand breaks that can be determined by the comet assay. After the lysis, the slides were washed (three times) using the enzyme buffer (40 mM HEPES–KOH, 0.5 mM Na_2_EDTA, 0.1 M KCl, 0.2 mg/mL BSA; pH 8) for 5 min each time. Next, agarose on slides was covered with a volume of 50 μL of buffer consisting of 1 U of Nth or hOGG1 or without the enzyme. Then, the slides were covered with cover glasses and were incubated for 30 min at 37 °C in a moist chamber. In the next step, the cover glasses were removed and the slides were placed in an electrophoresis unit^76,77^. DNA was allowed to unwind for 20 min in a solution consisting of 300 mM NaOH and 1 mM EDTA (pH > 13). We also performed analysis of oxidized DNA bases by determining the level of purine and pyrimidine oxidation in the positive control, which referred to the cells incubated with hydrogen peroxide at 20 μM for 15 min on ice and subsequently treated with the enzymes. The procedure was then carried out according to the comet assay alkaline version. We did not calibrate the enzymes. According to the recommendation contained in the BioLabs protocol in which our experiment based on, dilution of hOGG1 and Nth enzyme should be from 1: 102 to 1: 103 and from 1: 104 to 1: 105, respectively. It means that 50 μL of enzyme buffer with proper enzyme is equivalent of 0.08–0.8 U for hOOG1 and 0.05–0.5 U for Nth. Based on literature data^78^ we decided to use 1 U of each enzyme per gel, which guaranteed their use in excess (therefore, the calibration curve was not performed).

### Oxidation of H_2_DCFDA and HPF

After incubation with DBP, BBP, MBP or MBzP, the cells were centrifuged (600*g* for 10 min at 4 °C), suspended in PBS (final density 1 × 10^6^ cells/mL), and then incubated with H_2_DCFDA or HPF for 20 min at 37 °C in total darkness**.** In order to determine the production of total ROS, the fluorescence of the probe H_2_DCFDA was measured^79^. When H_2_DCFDA diffuses across the cellular membrane, it is hydrolyzed by membrane esterases to H_2_DCF. The increase in fluorescence of DCF (a marker of probe oxidation) was measured by a flow cytometer (LSR II Becton Dickinson) at excitation/emission wavelengths maxima of 488 nm and 530 nm, respectively. The final concentration of H_2_DCF in the sample was 5 μM. The analysis of 10,000 cells was performed^1^. We also used HPF to detect highly reactive oxygen species (mainly hydroxyl radical). The final concentration of HPF in PBMCs suspension was 4 μM. The increase in fluorescence intensity of oxidized form of HPF (a marker of probe oxidation) was measured by a flow cytometer (LSR II Becton Dickinson) at excitation/emission wavelengths maxima of 490 nm and 515 nm, respectively. The analysis of 10,000 cells was performed^1^.

### Kinetics of DNA repair

Control cells and the PBMCs treated with DBP, BBP, MBP or MBzP were washed and resuspended in fresh RPMI 1640 medium with L-glutamine preheated to 37 °C. Aliquots of the suspension were taken immediately (“time zero”) as well as 30 min, 60 min, 90 min and 120 min later. The samples were placed in an ice bath to stop DNA repair. Samples prepared in this way were measured in accordance with the procedure ,, Alkaline version ''. DNA repair was assessed by the extent of residual DNA damage detection at each time-point^1^.

### Statistical analysis

The results were shown as mean ± SD achieved from 5 individual experiments (5 blood donors). For each individual (donor), an experimental point was a mean value of 3 replications. We checked a normality of distribution using Shapiro–Wilk test as well as the homogeneity of variance by Brown-Fisher test. Multiply comparisons among group mean differences were analysed by one-way analysis of variance (ANOVA) followed by Tukey post hoc test. When p value was lower than 0.05, the difference was considered to be statistically significant. Statistical analysis was conducted using STATISTICA v. 13. software (StatSoft, Inc, Tulusa, USA)^70^.

## Supplementary Information

Supplementary Information.
