# Supplementary material for: Genotoxic risk assessment and mechanism of DNA damage induced by phthalates and their metabolites in human peripheral blood mononuclear cells
Source: Sci Rep. 2021 Jan 18;11:1658. doi: 10.1038/s41598-020-79932-5 (PMC7814068; doi:10.1038/s41598-020-79932-5)
Supplement: Supplementary file 1 — Supplementary Information. [file 41598_2020_79932_MOESM1_ESM.pdf]

Genotoxic risk assessment and mechanism of DNA damage induced by phthalates and their metabolites in human peripheral blood mononuclear cells

Paulina Sicińska, Katarzyna Mokra, Katarzyna Woźniak, Jaromir Michałowicz, Bożena Bukowska

Suplementarny materials:

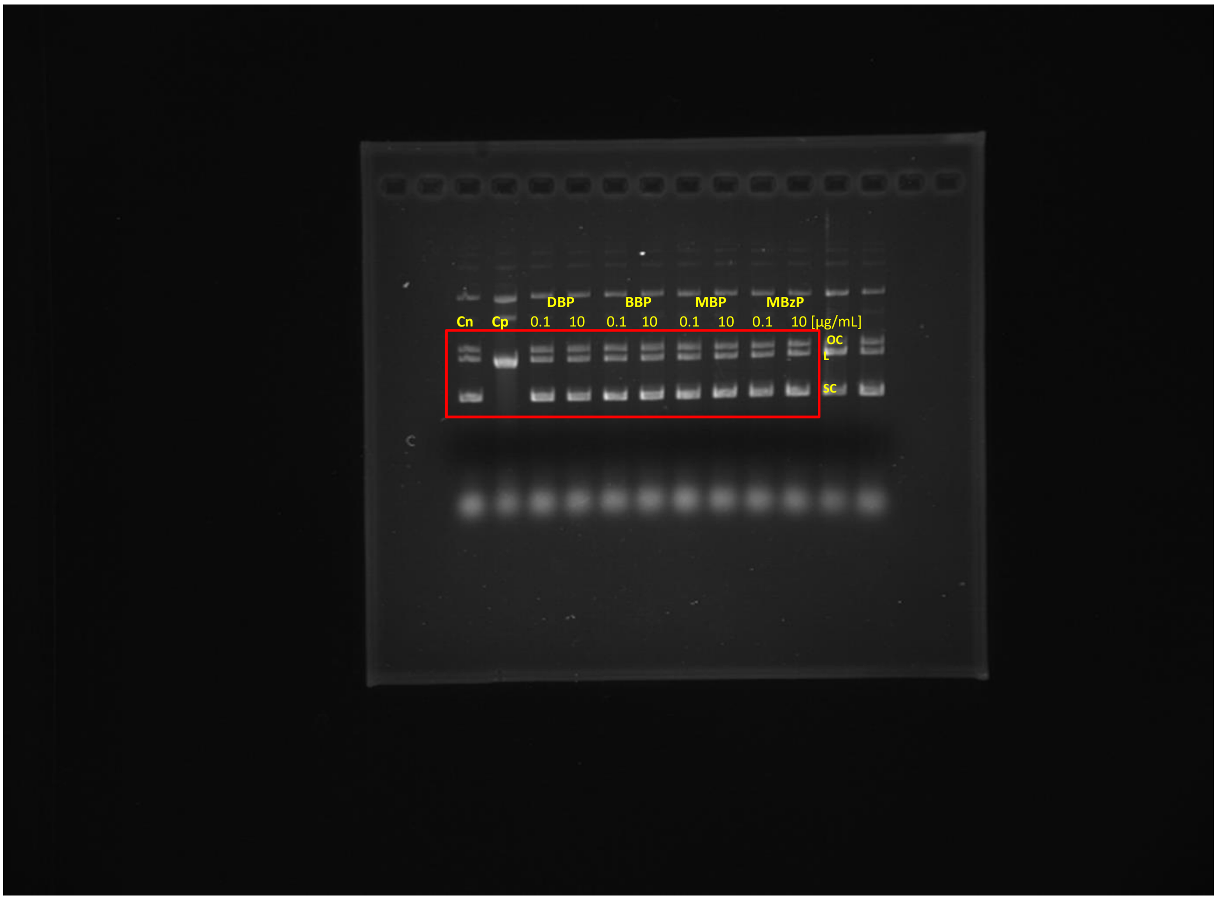

Fig. S1. Plasmid relaxation assay.

pUC19 plasmid DNA was resolved on a 1 % agarose gel, stained with ethidium bromide and visualized in UV light. Line 1 – negative control (Cn) (pUC19 plasmid); line 2 – positive control (Cp) (the plasmid was exposed to 200 µM H_2O_2 and 20 µM Fe^{+2} for 20 min on ice, Fenton reaction); lines 3-10 - pUC19 plasmid incubated with DDP, BBP, MBP, MBzP at indicated concentrations. Structural differences between supercoiled (SC), open circular (OC) and linear (L) forms of the plasmid accounted for their different electrophoretic mobility.
